# Vickybot, a chatbot for anxiety-depressive symptoms and work-related burnout

**DOI:** 10.1192/j.eurpsy.2023.301

**Published:** 2023-07-19

**Authors:** G. Anmella, M. Sanabra, M. Primé-tous, X. Segú, M. Cavero, R. Navinés, A. Mas, V. Olivé, L. Pujol, S. Quesada, C. Pio, M. Villegas, I. Grande, I. Morilla, A. Martínez-Aran, V. Ruiz, E. Vieta, D. Hidalgo-Mazzei

**Affiliations:** 1 Hospital Clínic de Barcelona; 2 Barcelona Supercomputing Center (BSC). Text Mining Technologies in the Health Domain., Barcelona, Spain

## Abstract

**Introduction:**

A significant proportion of people attending Primary Care (PC) have anxiety-depressive symptoms and work-related burnout and there is a lack of resources to attend them. The COVID-19 pandemic has worsened this problem, particularly affecting healthcare workers, and digital tools have been proposed as a workaround.

**Objectives:**

We present the development, feasibility and effectiveness studies of chatbot (Vickybot) aimed at screening, monitoring, and reducing anxiety-depressive symptoms and work-related burnout in PC patients and healthcare workers.

**Methods:**

User-centered development strategies were adopted. Main functions included self-assessments, psychological modules, and emergency alerts. (1) Simulation: HCs used Vickybot for 2 weeks to simulate different possible clinical situations and evaluated their experience. (3) Feasibility and effectiveness study: People consulting PC or healthcare workers with mental health problems were offered to use Vickybot for one month. Self-assessments for anxiety (GAD-7) and depression (PHQ-9) symptoms, and work-related burnout (based on the Maslach Burnout Inventory) were administered at baseline and every two weeks. Feasibility was determined based on the combination of both subjective and objective user-engagement Indicators (UEIs). Effectiveness was measured using paired t-tests as the change in self-assessment scores.

**Results:**

(1) Simulation: 17 HCs (73% female; mean age=36.5±9.7) simulated different clinical situations. 98.8% of the expected modules were recommended according to each simulation. Suicidal alerts were correctly activated and received by the research team. (2) Feasibility and effectiveness study: 34 patients (15 from PC and 19 healthcare workers; 77% female; mean age=35.3±10.1) completed the first self-assessments, with 34 (100%) presenting anxiety symptoms, 32 (94%) depressive symptoms, and 22 (64.7%) work-related burnout. Nine (26.5%) patients completed the second self-assessments after 2-weeks of use. No significant differences were found for anxiety [t(8) = 1.000, p = 0.347] or depressive [t(8) = 0.400, p = 0.700] symptoms, but work-related burnout was significantly reduced [t(8) = 2.874, p = 0.021] between the means of the first and second self-assessments. Vickybot showed high subjective-UEIs, but low objective-UEIs (completion, adherence, compliance, and engagement).

**Conclusions:**

The chatbot proved to be useful in screening the presence and severity of anxiety and depressive symptoms, in reducing work-related burnout, and in detecting suicidal risk. Subjective perceptions of use contrasted with low objective-use metrics. Our results are promising, but suggest the need to adapt and enhance the smartphone-based solution in order to improve engagement. Consensus on how to report UEIs and validate digital solutions, especially for chatbots, are required.

**Disclosure of Interest:**

None Declared

